# IL-17-related signature genes linked to human necrotizing enterocolitis

**DOI:** 10.1186/s13104-021-05489-9

**Published:** 2021-03-04

**Authors:** Éric Tremblay, Emanuela Ferretti, Corentin Babakissa, Karolina Maria Burghardt, Emile Levy, Jean-François Beaulieu

**Affiliations:** 1 Laboratory of Intestinal Physiopathology, Faculté de médecine et sciences de la santé, Université de Sherbrooke, Main Building Room 9425, Sherbrooke, QC J1H 5N4 Canada; 2 Division of Neonatology, Department of Pediatrics, CHEO, Ottawa, ON Canada; 3 Department of Pediatrics, Faculté de médecine et sciences de la santé, Université de Sherbrooke, Sherbrooke, QC Canada; 4 Division of Gastroenterology, Hepatology & Nutrition, CHEO, Ottawa, ON Canada; 5 Department of Nutrition, Centre de Recherche, CHU Sainte-Justine, Université de Montréal, Montréal, QC Canada

**Keywords:** Human intestine, Preterm newborn, Transcriptomics, Gene expression, Interleukin-17

## Abstract

**Objective:**

Necrotizing enterocolitis (NEC) is the most frequent life-threatening gastrointestinal disease experienced by premature infants in neonatal intensive care units all over the world. The objective of the present study was to take advantage of RNA-Seq data from the analysis of intestinal specimens of preterm infants diagnosed with NEC. Function enrichments with Gene Ontology and the Kyoto Encyclopedia of Genes and Genomes were used to analyse previous data in order to identify biological and functional processes, which could provide more insight into the pathogenesis of NEC in infants.

**Results:**

Gene set enrichment analysis indicated that the most significant biological pathways over-represented in NEC neonates were closely associated with innate immune functions. One of the striking observations was the highly modulated expression of inflammatory genes related to the IL-17 pathway including such as pro-inflammatory cytokines (CXCL*8*), chemokines (*CXCL5 and CXCL10*) and antimicrobials (*DEF5A, DEF6A, LCN2, NOS2*) in the intestine of neonates diagnosed with NEC. Interestingly, the increase in IL-17 expression appeared to be under the IL-17F form, as reported in Crohn's disease, another inflammatory bowel disease. Further investigation is thus still needed to determine the precise role of IL-17F and its downstream targets in NEC.

## Introduction

Necrotizing enterocolitis (NEC) is the most common life-threatening condition of premature infants occurring in neonatal intensive care units [1–3]. This gastrointestinal disease is characterized by severe intestinal inflammation, intestinal necrosis and high morbidity [4]. The infants who survive NEC are at a higher risk for developing short bowel syndrome, impaired growth and neurodevelopmental outcomes [5, 6]. Several epidemiological investigations have suggested that preterm birth, enteral feeding and abnormal bacterial colonization are likely to play major roles in the pathogenesis of NEC [7, 8]. However, the precise mechanisms leading to the development of NEC remain to be elucidated.

One of the greatest challenges for neonatologists is to identify early reliable clinical signs and symptoms of NEC [1, 2]. In this context, the ahead of time clinical manifestations of NEC are relatively nonspecific and at diagnosis NEC is often already at an advanced stage due first to the initially insidious condition and then to the fierce progression of the disease [9, 10]. One strategy to prevent or treat NEC would be to develop an early diagnostic tool allowing the identification of preterm infants either at risk of developing NEC or at the onset of symptoms to aid in the diagnostic dilemma and treatment.

Recently, we used the high-throughput sequencing of RNA transcripts (RNA-Seq) approach to determine the complete gene expression profiles of ileal specimens, which were resected from preterm infants diagnosed with NEC vs non-NEC conditions [11]. This recently important work has led to the identification of potential biomarkers for the prediction of the disease. The objective of the present study was to provide new insights into the molecular mechanisms of NEC pathophysiology by conducting function enrichment analyses with Gene Ontology (GO) and the Kyoto Encyclopedia of Genes and Genomes (KEGG). Our analysis revealed that immune and inflammatory responses were strongly modulated in the small intestine of neonates with NEC, supporting the finding that NEC development is related to the immaturity of the intestinal mucosa challenged by microbial dysbiosis [12, 13]. Interestingly, we also observed that several interleukin-17 (IL-17) target genes, including chemokines and antimicrobial proteins, were strongly modulated in the small intestine of neonates with NEC. Pro-inflammatory IL-17 has been linked to the pathogenesis of diverse autoimmune and chronic inflammatory diseases while being essential for host defense against microbial colonization [14, 15]. Our results suggest a role for IL-17 signaling, specifically IL-17F, in the development of NEC.

## Main text

### Materials and methods

#### Study population and informed consent

As detailed in the previous report [11], the present collaborative study recruited premature infants from neonatal intensive care units at the Centre Hospitalier Universitaire de Sherbrooke (Sherbrooke, QC, Canada), Erasmus MC-Sophia Children’s Hospital (Rotterdam, The Netherlands), Children’s Hospital of Eastern Ontario (Ottawa, ON, Canada) and Hôpital Pierre Zobda-Quitman (Fort-de-France, Martinique), between October 2008 and May 2013. After the approval of the Institutional Review Committee for the use of human material in each center, written informed consent from parents or guardians was obtained for each patient.

Premature infants who had undergone bowel resection were eligible for the study. The diagnosis and staging of NEC were based according to the Bell's criteria [9]. Freshly resected intestinal specimens taken from the ileum were conserved in RNAlater (Ambion) before RNA extraction. Preterm patients who had undergone bowel resection for stage III acute NEC were referred to as NEC, and preterm patients who had undergone resection for diseases other than NEC were included as control patients (CTRL) as detailed in [11].

#### Sample preparation and RNA sequencing

The procedures for sample preparation, library preparation and sequencing, and data analysis were performed as previously described by Tremblay et al. [11].

#### Functional pathway enrichment analysis

The bioinformatics software DAVID 6.8 (https://david.ncifcrf.gov/) [16] was used to perform Gene Ontology (GO) analysis and KEGG pathways enrichment analysis of differentially expressed genes (DEGs) identified in the pathogenesis of NEC.

#### Data validation by quantitative polymerase chain reaction (qPCR)

All qPCR reactions were performed as previously described [11]. The genes investigated were chemokine (C-X-C motif) ligand 8 (*CXCL8*) and 10 (*CXCL10*), alpha-defensin 5 (*DEFA5*) and 6 (*DEFA6*), interleukin-17A (*IL-17A*) and IL1-7F (*IL-17F*), interleukin 6 (*IL6*), lipocalin 2 (*LCN2*), nuclear factor kappa B subunit 1 (*NFKB1*), regenerating islet-derived 3 alpha (*REG3A*), ribosomal protein lateral stalk subunit P0 (*RPLP0*), ribosomal protein 23 (*RPS23*), and tumor necrosis factor (*TNF*). Primers (listed in Additional file 1: Table S1) were generated using the primer formation software Primer3 (http://bioinfo.ut.ee/primer3). Differences in gene expression were evaluated by comparing the expression of control and NEC samples using the equation R = (E_target_)^∆Ct target^/(E_reference_)^∆Ct reference^) [17]. Samples were normalized to a set of 2 reference genes, *RPLP0* and *RPS23* [18, 19].

### Results

#### RNA-Seq analysis and identification of DEGs

The data for RNA-Seq analysis on ileum of preterm infants with NEC vs without NEC (CTRL) have been deposited at the National Center for Biotechnology Information's Gene Expression Omnibus and are accessible through GEO Series accession number GSE64801 (https://www.ncbi.nlm.nih.gov/geo/query/acc.cgi?acc=gse64801). In total, 804 DEGs were identified (p < 0.05), 383 upregulated and 421 downregulated [11].

#### GO and KEGG pathway analyses in NEC samples

**Fig. 1:**
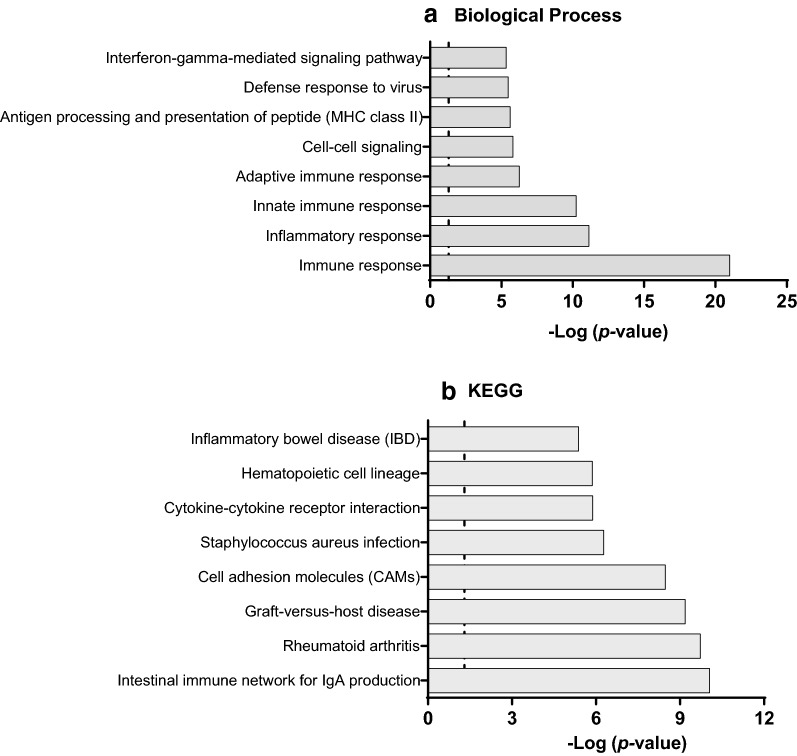
Functional pathways in NEC samples. The negative logarithm of *p*-values (Fisher's test) calculated for the 8 most significant pathways overrepresented in small intestinal NEC samples compared to control non-NEC samples for GO Biological process (**a**) and KEGG (**b**) pathways. Dotted line: -Log 0.05 corresponds to 1.3

**Fig. 2:**
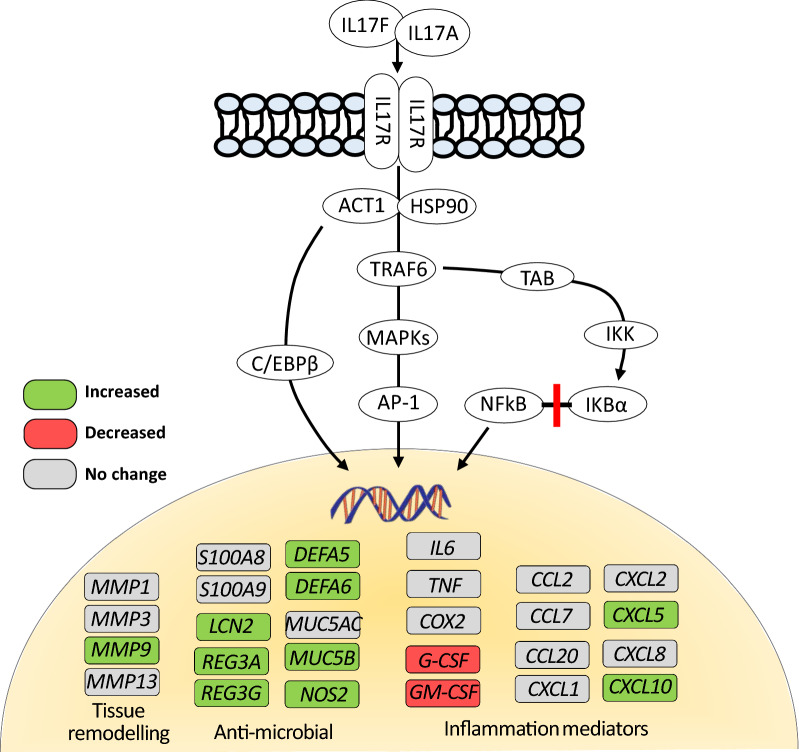
Association of DEG identified in NEC samples and IL-17 target genes. IL-17 receptor (IL-17R) can bind to IL-17A, IL-17F or IL-17A/F to activate various signaling pathways leading to the regulation of IL-17 targets genes involved in tissue remodeling and anti-microbial activity as well as mediators of inflammation as adapted from [15, 20]. Genes identified as being significantly modulated in NEC samples compared with control non-NEC samples are labeled in green and red while those for which no significant differences were notes are labeled in grey

In the previous study, IPA analysis revealed an impaired immune response in the intestine of NEC neonates where several related pathways and biological functions were found to be altered in the samples of NEC infants [11]. To gain more insight in the function pattern of the DEGs obtained previously, GO and KEGG pathway analyses were conducted by DAVID. Among the 151 GO "Biological Process" terms enriched in DEGs, the top 8 terms, which were selected based on their highest *p* values, were highly associated with innate and adaptive immune as well as inflammatory responses (Fig. 1a). The same relationship with the immune responses was also noted with KEGG analyses (Fig. 1b). Intriguingly, a further detailed analysis of the DEGs revealed that many of those genes were highly associated with the signature target genes of IL-17 (Fig. 2), suggesting a role for this cytokine in the pathogenesis of NEC. Among these IL-17 target genes, changes in expression of pro-inflammatory cytokines, chemokines, antimicrobials and matrix metalloproteinase were identified.

#### Validation of the gene expression profile in NEC samples

**Fig. 3:**
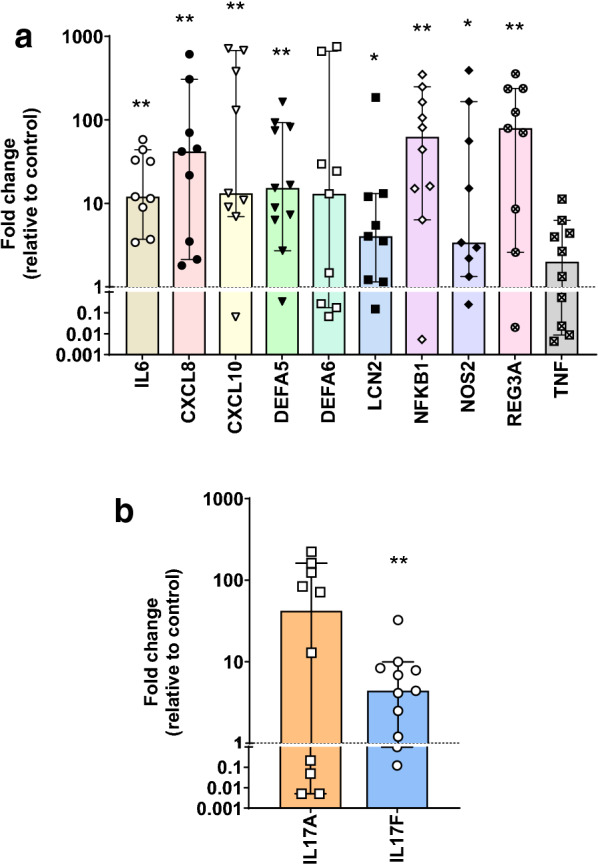
Differential expression of IL-17-related genes assessed in intestinal NEC and non-NEC control samples by real-time qPCR. Transcript levels of IL-17 target genes (**a**) and *IL17A and IL17F* transcripts (**b**) in NEC relative to control. Results are expressed as a scatter dot plot showing individual data and as median with 95% confidence interval. * and ** are *p* < 0.05 and *p* < 0.01, respectively, using the one sample Wilcoxon test

To further investigate the gene expression profile identified in RNA-Seq analyses, we used qPCR to test representative DEGs known to be involved in IL-17 signaling in NEC samples as depicted in Fig. 2. As shown in Fig. 3a, qPCR analyses confirmed the upregulation of many of the IL-17 downstream genes in NEC samples. Incidentally, transcript expression of the two main IL-17 cytokines, IL-17A and IL-17F was also investigated and revealed a significant induction of IL-17F in NEC samples (Fig. 3b) confirming alterations in the IL-17 signature gene profile in the intestinal samples of patients with NEC.

### Discussion

NEC is a complex and a potentially fatal disease that remains a major cause of morbidity in neonates. Although significant effort has been made on the further understanding of this disease, no effective treatment or further diagnostic tools have been discovered [3]. In the present study, we performed GO and KEGG pathway analyses on RNA-seq data that was previously done with the aim to further deepen our knowledge of the molecular mechanisms underlying NEC pathogenesis. GO and KEGG pathway analyses showed strong enrichment in genes involved in innate and adaptive immune responses, consistent with a disproportionate inflammatory response following an exposure to luminal microorganisms [11]. Indeed, excessive inflammatory response and overexpression of proinflammatory cytokines are the main pathophysiological characteristics of NEC that contribute to damage the intestinal epithelium [13]. Moreover, by reviewing in detail all enriched genes in these pathways, we highlighted several target genes of the IL-17 pathway [20], which were modulated in NEC, suggesting a role for IL-17 signaling in the pathophysiology of this severe inflammatory disease.

The IL-17 family of cytokines consists of six structurally related proteins, IL-17A through IL-17F [15]. IL-17A and IL-17F are the most related members and they are usually secreted as homodimers or heterodimers by various innate immune cells [21] including those residing in the gut [22]. One of the major functions of IL-17 is to provide a protective inflammatory response to the host against pathogens [23–25]. However, despite the protective effects of IL-17 during infection, there is emerging evidence that dysregulated IL-17 expression could worsen the gravity of some diseases [26, 27], namely NEC [28, 29]. It is noteworthy that IL-17 exerts only modest activity by its own, its immunomodulatory role arising from synergistic action with other cytokines [24]. In preterm infants, IL-17 level was found to be increased after red blood cell transfusion that could lead to a higher risk of developing NEC [30]. Moreover, an increased IL-17 level has been related to impairment of enterocyte tight junctions and increased epithelial cell apoptosis through Toll-like receptor 4-dependent Th17 polarization in mouse and human NEC models [31] consistent with its key role in host defence [24]. Indeed, as reviewed recently, although evidence for the involvement of adaptative immunity in NEC initiation and perpetuation is still insufficient, the potential for a pathogenic role of Th17 responses is pointed out [25]. In our study, we showed that the transcript expression of IL-17F, but not IL-17A, is upregulated in the intestine of premature infants diagnosed with NEC. Distinct roles between IL-17A and IL-17F have been previously demonstrated in IL-17F-deficient mice [32, 33]. Upregulated IL-17F expression was also reported in intestinal samples obtained from patients with active Crohn's disease [34]. In the experimental colitis model induced by DSS, IL-17F^−/−^ mice were protected, whereas IL-17-knockout mice showed a more severe condition [32]. In addition, a recent study showed that a potential genetic variant of IL-17F, but not IL-17A, was associated with an increased severity of NEC [35]. Taken together, these observations support a potential involvement for the upregulated expression of IL-17F in the development of NEC. Based on the similarity between NEC and Crohn's disease [11], one may speculate that as proposed for Crohn's disease [36], the development of specific inhibitors for IL-17F could become a potential option for treating NEC although it is important to consider that other organs such as the lung or the brain could also be affected by IL-17 blockade [37, 38].

Upregulation of IL-17 controls the expression of inflammatory genes in the majority of non-hematopoietic cells [39]. The trademark of IL-17 signaling is induction of pro-inflammatory cytokines, chemokines, antimicrobial proteins and inflammatory effectors, to maintain mucosal barrier integrity against infections [20, 40]. Among the IL-17 signature genes, we have found the transcription factor *NFKB1*, chemokines such as *CXCL8, CXCL10* as well as a plethora of antimicrobial molecules or regulators (*DEFA5, LCN2, NOS2, REG3A*) to be upregulated in NEC intestinal samples as compared to control. These observations suggest that IL-17 could be involved in a protective mucosal reaction towards pathogenic microorganisms in the intestine of NEC infants. Unexpectedly, we observed that expression levels of IL-17-related pro-inflammatory cytokines (*CSF2, CSF3,* and *TNF*) were either downregulated or unchanged in NEC tissues. In the early phase of infection, these cytokines have been reported to be involved in neutrophil recruitment for an effective innate immunity and rapid control against pathogens [41, 42]. One possible explanation of this discrepancy could be the time lapse between early induction of these cytokines and the resection of tissue that occurred at the late phase of the disease.

In conclusion, this study has led to the identification of a characteristic IL-17 core gene signature in the pro-inflammatory intestinal microenvironment of the premature infant with NEC. Furthermore, it appears that it is IL-17F, rather than IL-17A, which is consistently involved in the pro-inflammatory cascade in neonates affected with NEC. However, further investigations need to be undertaken to validate the role of IL-17F in the development of NEC at the functional level.

## Limitations

Intestinal samples from premature infants diagnosed with NEC are difficult to obtain [43]. It is noteworthy that the small cohort of control and NEC patients represents a limitation in interpreting the results. A larger cohort which could also include NEC patients at earlier stages would help to better document the role of IL-17 in NEC initiation as well as increase the statistical power to better detect differentially expressed genes.

## Supplementary Information

**Additional file1: Table S1.** Primers used for qPCR.

## Data Availability

The datasets used during the current study are available through GEO Series accession number GSE64801. The datasets analyzed during the current study are available from the corresponding author on reasonable request.

## Abbreviations

CTRL: Control
DEG: Differentially expressed genes
GO: Gene ontology
IL-17: Interleukin-17
KEGG: Kyoto Encyclopedia of Genes and Genomes
NEC: Necrotizing enterocolitis
qPCR: Quantitative polymerase chain reaction
RNA-Seq: High-throughput sequencing of RNA transcripts
