# Supplementary material for: IL-17-related signature genes linked to human necrotizing enterocolitis
Source: BMC Res Notes. 2021 Mar 4;14:82. doi: 10.1186/s13104-021-05489-9 (PMC7934396; doi:10.1186/s13104-021-05489-9)
Supplement: Supplementary file 1 — Additional file1: Table S1. Primers used for qPCR. [file 13104_2021_5489_MOESM1_ESM.docx]

| **Table S1. Primers used for qPCR** | |  |  |
| --- | --- | --- | --- |
| **Gene symbol** | **Sense primer** | **Antisense primer** | **Accession No.** |
| CXCL8 | 5'-GTGCAGTTTTGCCAAGGAGT-3' | 5'-CTCTGCACCCAGTTTTCCTT-3' | NM_000584 |
| CXCL10 | 5'-CTGTACGCTGTACCTGCATCA-3' | 5'-TTCTTGATGGCCTTCGATTC-3' | NM_001565 |
| DEFA5 | 5’-AAGCAGTCTGGGGAAGACAA-3’ | 5’-TGAATCTTGCACTGCTTTGG-3’ | NM_021010 |
| DEFA6 | 5'-GCAGGCAAAAGCTTATGAGG-3' | 5'-TCTGCAATGGCAAGTGAAAG-3' | NM_001926 |
| IL17A | 5'-ACCAATCCCAAAAGGTCCTC-3' | 5'-GGGGACAGAGTTCATGTGGT-3' | NM_002190 |
| IL17F | 5'-TGAAGCTTGACATTGGCATC-3' | 5'-TTCCTTGAGCATTGATGCAG-3' | NM_052872 |
| IL6 | 5'-AGGAGACTTGCCTGGTGAAA-3' | 5'-CAGGGGTGGTTATTGCATCT-3' | NM_000600 |
| LCN2 | 5'-TCACCTCCGTCCTGTTTAGG-3' | 5'-CGAAGTCAGCTCCTTGGTTC-3' | NM_005564 |
| NFKB1 | 5'-CCTGGATGACTCTTGGGAAA-3' | 5'-TCAGCCAGCTGTTTCATGTC-3' | NM_003998 |
| NOS2 | 5'-CATCCTTGCATCCTCATCG-3' | 5'-CACCCAAACACCAAGGTCAT-3' | NM_000625 |
| REG3A | 5'-TGTCACCAAAATCCTGGACA-3' | 5'-GGATTTCTCTCCCATGCAAA-3' | NM_002580 |
| RPLPO | 5'-TCGACAATGGCAGCATCTAC-3' | 5'-GCCTTGACCTTTTCAGCAAG-3' | NM_001002 |
| RPS23 | 5’-AGGAAGTGTGTAAGGGTCCAGC-3’ | 5’-CACCAACAGCATGACCTTTGCG-3’ | NM_001025 |
| TNF | 5'-AGCCCATGTTGTAGCAAACC-3' | 5'-ATGAGGTACAGGCCCTCTGA-3' | NM_000594 |
